# Dietary impact of a plant-derived microRNA on the gut microbiome

**DOI:** 10.1186/s41544-020-00053-2

**Published:** 2020-08-11

**Authors:** Jennifer K. Spinler, Numan Oezguen, Jessica K. Runge, Ruth Ann Luna, Vivekanudeep Karri, Jian Yang, Kendal D. Hirschi

**Affiliations:** 1 Texas Children’s Microbiome Center, Department of Pathology, Texas Children’s Hospital, Houston, TX, USA.; 2 Department of Pathology & Immunology, Baylor College of Medicine, One Baylor Plaza, 1102 Bates Ave, Houston, TX 77030, USA.; 3 Rice University, Houston, TX, USA.; 4 Pediatrics-Nutrition, Children’s Nutrition Research, Baylor College of Medicine, 1100 Bates Ave, Houston, TX 77030, USA.

**Keywords:** Microbiome, miRNA, Transgenic plant, Mice

## Abstract

**Background:**

Global estimations of 4 billion people living on plant-based diets signify tremendous diversity in plant consumption and their assorted miRNAs, which presents a challenging model to experimentally address how plant-based miRNAs impact the microbiome. Here we establish baseline gut microbiome composition for a mouse model deficient in the specific mammalian miR-146a shown to alter gut microbiomes. We then asses the effect on the gut microbiome when miR-146a-deficient mice are fed a transgenic plant-based diet expressing the murine-derived miR-146a. Mice deficient in miR-146a were maintained either on a baseline diet until 7 weeks of age (day 0) and then fed either vector or miR-146a-expressing plant-based diets for 21 days. The gut microbiomes of mice were examined by comparing the V4 region of 16S rRNA gene sequences of DNA isolated from fecal samples at days 0 (baseline diet) and 21 (vector or miR-146a expressing plant-based diets).

**Results:**

Beta-diversity analysis demonstrated that the transition from baseline chow to a plant-based diet resulted in significant longitudinal shifts in microbial community structure attributable to increased fiber intake. Bipartite network analysis suggests that miR-146a-deficient mice fed a plant diet rich in miR-146a have a microbiome population modestly different than mice fed an isogenic control plant diet deficient in miR-146a.

**Conclusion:**

A mouse diet composed of a transgenic plant expressing a mouse miR-146a may fine tune microbial communities but does not appear to have global effects on microbiome structure and composition.

## Background

Beneficial effects of plant-based diets have been linked to intestinal health through the promotion of greater diversity and even distribution of gut microbiota [1, 2], yet the beneficial bioactive components in plant-based diets are largely unknown. Plants, animals, and prokaryotes all possess microRNAs (miRNAs) which are evolutionarily conserved, single-stranded, noncoding RNAs that regulate gene expression through sequence-specific gene repression. Plant-based diets contain thousands of miRNAs with bioactive potential to impact the gut microbiome to the consumer’s advantage.

Recent advances in microbiome science suggest that miRNAs can impact host health by modulating the gut microbiota [3–9]. It is known that mammalian gut epithelial cells excrete miRNAs into the gut lumen, making endogenous miRNAs normal components of fecal content that regulate gut-associated bacterial gene transcripts [10], setting precedence for other miRNA-directed interkingdom communication. Exosomes (lipid-based nanoparticles) encapsulate miRNAs and are shed from almost all cell types to interact with specific target cells. Recently, the gut microbiome has been implicated in childhood malnutrition [11, 12], and cooperative diet-microbe interactions could be important aspects of malnutrition-related deficiencies targeted by therapeutic foods. The World Health Organization recommends that all malnourished children be treated with therapeutic foods; however, the health-promoting components of many foods have not been identified.

Plant diets are associated with intestinal health and promoting the development of a diverse and stable microbial ecosystem [13]. Plants use exosome-like nanoparticles (ELNs) to communicate with microbes and fungi through the transport of various lipids, proteins and RNAs. Recently, a model has been proposed that each plant species has unique ELNs which differentially modulate bacteria within the gut [14]. The novelty in plant-based miRNA and gut microbiome interactions is that bioactive plant-derived miRNAs have the potential to influence microbiome function and positively impact host health without the requirement of direct uptake into the host’s circulation and transport to distal tissues. The challenge is to design sensitive biological reagents to test the hypothesis that dietary plant miRNAs directly impact the microbiome.

Estimations of 4 billion people living on plant-based diets world-wide signify tremendous diversity in plant consumption and their assorted miRNA cargos, making the ability to address how plant-based miRNAs impact the microbiome quite challenging. This work addresses the impact of dietary plant-based miRNAs on the gut microbiome using transgenic plant diets in a mouse model deficient in miR-146a (Supplemental Figure 1). Animals lacking miR-146a have both impaired intestinal health and an altered gastrointestinal (GI) microbiome [15]. We focus on the role of miR-146a in intestinal homeostasis using plant-based diets expressing either empty vector control or murine miR-146a to feed miR-146a-deficient mice. In this study, we tested the hypothesis that a plant-based dietary miRNA can alter the microbiome composition of the consumer.

## Results

### Establishing miR-146a-associated differences in murine gut microbiomes

**Fig. 1:**
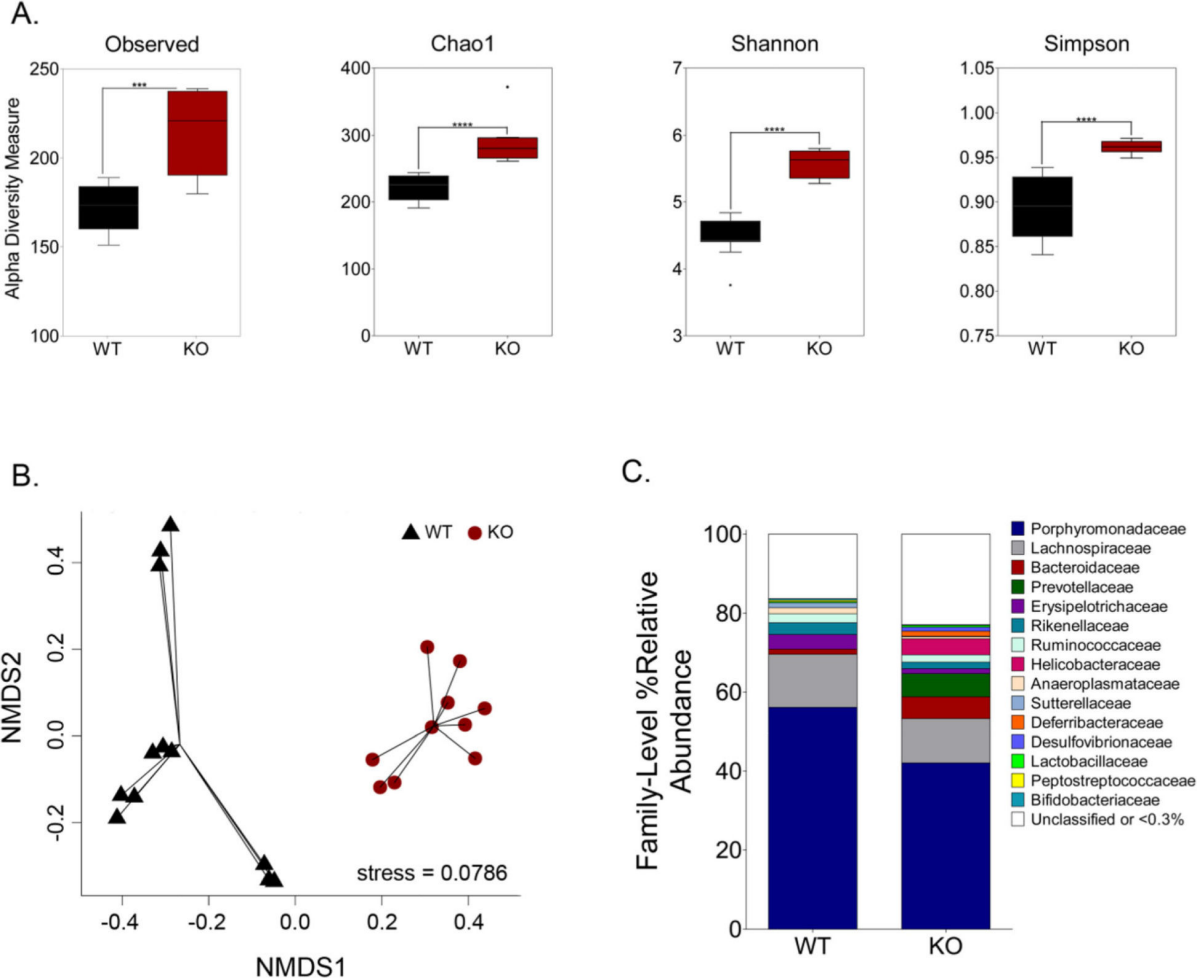
Distinct gut microbiome community structure differentiates WT and KO mice on chow diets. Analysis of 16S rRNA gene sequence data generated from fecal samples. **a** The lack of miR-146a expression in KO mice (dark red boxes) results in increased species richness and evenness when compared to WT mice (black boxes) as measured by the following alpha-diversity indices: observed species, Chao1, Shannon-diversity, and Simpson-diversity. Significance was determined by the Mann-Whitney U test; ***p < 001, ****p < 0001. **b** The Bray-Curtis dissimilarity measure was used to evaluate pairwise relationships between samples and data were plotted with nonmetric multidimensional scaling (NMDS). Each point represents an individual animal. **c** Taxonomic summary of percent relative abundance shows differential distribution of OTUs at the family-level phylogeny between WT and KO mice

**Fig. 2:**
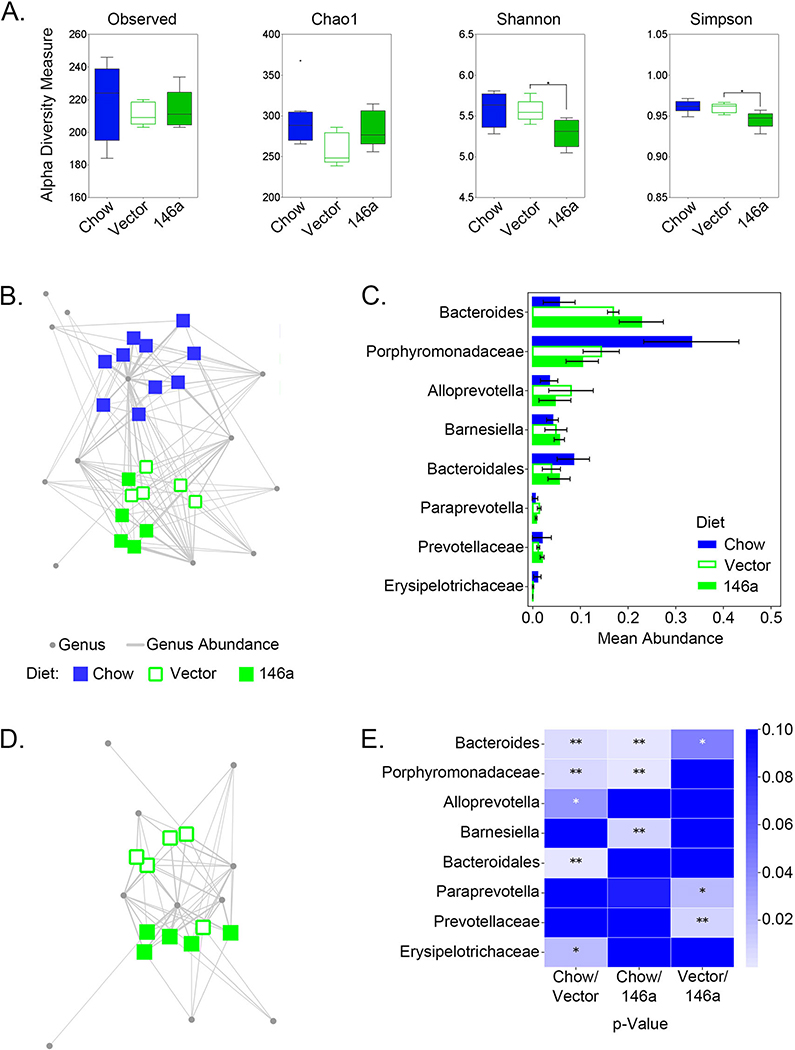
Characterization of gut microbiomes from mice on chow or plant-based precision diets. **a** Observed species, Chao1, Shannon-diversity, and Simpson-diversity measures of microbiome sequence data from KO mice fed chow, vector, or miR-146a diets. Significance (*p < 0.05) was determined by the Mann-Whitney U test. **b** Bipartite network profile of mice on baseline chow diet (blue squares), plant-based vector diet (open green squares), and plant-based miR-146a diet (full green squares). **c** Bar graph of the mean abundance of genera with means > 0.01 abundance in at least one diet group. Error bars represent standard deviation (95% confidence). **d** Bipartite network comparisons of mice on vector (open green squares) and miR-146a (closed green squares) diets. Gray circles and edges represent the genera and genera-OTU abundances, respectively, for both (**b**) and (**d**). **e** Heatmap visualization of paired *p*-values from t-tests comparing 1) chow vs vector, 2) chow vs miR-146a, and 3) vector vs miR-146a diets for the genera shown in (**c**)

To establish baseline gut microbiome compositions for our study, we used a mouse model deficient in the specific mammalian miR-146a miRNA [16, 17]. Previous studies have shown gut microbiomes of mice deficient in miR-146a are distinct from that of wild-type mice [18]. We maintained 7-week-old C57BL/6 wild-type (WT) and miR-146a^−/−^ (KO) mice on regular chow diets for 30 days (*n* = 10/group), collected fecal samples, and sequenced the V4 variable region of 16S rRNA genes from DNA isolated from fecal specimens. No distinct differences between WT and KO mice were noted in health, weight, or behavior during these 30 days. Both richness and evenness in mean species diversity (alpha diversity) of the gut microbiota was significantly increased in KO mice when compared to WT. Species richness, as measured by both the observed species and Chao1 indices, indicate that ~ 25% more operational taxonomic units (OTUs, clustered at > 97% sequence identity) are present in mice not expressing miR-146a (Fig. 1a). Additionally, the absence of miR-146a results in an approximate 25% increase in species evenness as measured by Shannon and Simpson diversity indices that take into account both the number and abundance of OTUs (Fig. 1a). Overall differences in microbial community composition were evaluated using Bray-Curtis dissimilarities and visualized through nonmetric multidimensional scaling (NMDS) (Fig. 1b). Statistical testing of these differences using analysis of similarities (ANOSIM) demonstrated that the gut microbiomes of KO mice were significantly different from those of WT (*R* = 0.796, *p* < 0.001) (Fig. 1b). A comparison of family-level relative abundances highlighted that KO mice varied in OTUs representing a broad range of bacterial families and were specifically increased in OTUs representing the *Lactobacillaceae* (34%), *Bacteroidaceae* (77%), *Prevotellaceae* (> 99%), and *Helicobacteraceae* (> 99%) families (Fig. 2c). These results provided a baseline for us to study the microbiomes of WT and KO mice on chow diets and confirm that the KO mice have significantly altered gut microbiomes compared to their WT counterparts, further substantiating the link between miRNA expression and microbiome modulation.

### Analysis of vector control and miR-146a plant diets

Focusing on the role of miR-146a in intestinal homeostasis through microbiome modulation [15, 18], we hypothesized that the gut microbiome composition of miR-146a-deficient mice may be influenced by a transgenic plant-based diet expressing the murine-derived miR-146a (Supplemental Figure 1). It was unknown whether the expression or lack thereof of mir-146a would inadvertently alter nutrient content [19, 20] of the plants. Therefore, we utilized the computational sequence prediction tool psRNATarget [21] to identify putative spurious plant mRNA targets of miR-146a that could impact nutritional composition. Six potential Arabidopsis mRNA targets of miR-146a were identified (Supplemental Table 1), none of which appeared to be directly related to genes impacting nutrient levels. However, these predictions are often experimentally difficult to validate [22] making it necessary to analyze the macronutrient content of our diets. We cultivated isogenic Arabidopsis plants expressing either empty vector as a control (vector) or the murine miR-146a miRNA (146a) and mixed plant material with chow and water 1: 2:2 w/w/w to prepare the diets [23]. Diet composition (vector:chow and 146a:chow) was evaluated for moisture, protein, crude fat, insoluble dietary fiber, soluble dietary fiber, total fiber, and ash content to ensure no off-target effects of expressing miR-146a exist. This analysis revealed no significant differences in any of the measured parameters for either the vector or 146a diet (Table 1).

### Effects of dietary miR-146a on the gut microbiomes of miR-146a-deficient mice

The plant diets characterized above were used in murine feeding studies to test the hypothesis that gut microbiome composition of miR-146a-deficient mice may be influenced by providing miR-146a in trans through a transgenic plant-based diet expressing the murine-derived miR-146. Mice were initially maintained on a regular chow diet for 30 days (*n* = 10), then transitioned at 7 weeks of age (day 0) to either vector or 146a plant-based diets for 21 days (*n* = 5/group). Plant-based diets consisted of 5 g of either vector or 146a plants containing 1 g of dried plant material (the equivalent of 3.3 g fresh plant tissue per mouse daily) [24]. Fecal samples were collected from each mouse at day zero (prior to starting the plant-based diets) and at day 21. No distinct differences in health, weight, or behavior were noted between mouse diet groups during the length of the experiment. Histology of intestinal epithelial specimens of KO mice on a vector diet were compared to those of KO mice on a 146a diet and showed no significant differences in leukocyte infiltration, crypt hyperplasia, or villus blunting between miR-146a deficient mice on either vector or 146a diets at day 21 (Supplemental Figure 2).

### Plant fiber content drove gut microbiome changes in mir-146a-deficient mice

The effect of our transgenic plant-based diet expressing the murine-derived miR-146a on the gut microbiome composition of miR-146a-deficient mice was examined by comparing the V4 region of 16S rRNA gene sequences of DNA isolated from fecal samples at days 0 (baseline chow diet) and 21 (vector or 146a plant-based diets). While alpha-diversity measures showed no significant differences in species richness or evenness in microbiomes of KO mice on chow vs plant diets (Fig. 2a), beta-diversity analysis demonstrated that the transition from chow to a plant-based diet resulted in coordinated longitudinal shifts in microbial community structure. Genera abundance networks showed significant shifts (ANOSIM, *R* = 0.8098, *p* < 0.001) from baseline at day 0 (blue squares) upon feeding plant-based diets for 21 days (green squares; Fig. 2b). These shifts were mainly driven by a significant increase in the genera *Bacteroides* and *Alloprevotella* and a decrease in members of the families *Porphyromonadaceae* and *Erysipelotrichaceae* and a member of the order *Bacteroidales* (Fig. 2c). *Bacteroides* and *Alloprevotella* genera are members of the *Bacteroidetes* phylum and communities rich in members of this phylum have been associated with diets rich in starch, fiber and plant protein [25]. These changes are consistent with an increase in dietary fiber provided by our plant-based diet, and further confirm our model as a valid system for studying plant-based dietary changes in GI microbiota.

Additionally, our bipartite network analysis suggests that KO mice fed a plant diet rich in miR-146a have a microbiome population subtly different than mice fed an isogenic vector control plant diet (Fig. 2d). As one might expect, the changes in community structure between isogenic plant diets differing in the expression of a single miRNA were modest and not statistically significant (*R* = 0.304, *p* = 0.075), however, sub-populations of bacteria may be responsive to the dietary miRNAs. While alpha-diversity indicated no difference in species diversity between microbiomes associated with either the vector or 146a diet at day 21, Shannon and Simpson diversity metrics showed microbiomes established on the 146a diet had a slight yet significant (*p* < 0.05) decrease in even species distribution than those on vector diets (Fig. 2a). The 146a diet contributed to modest changes in microbiome populations driven by increased abundances in OTUs representing *Bacteroides* and the *Prevotellaceae* family, both members of the phylum Bacteroidetes (Fig. 2c and e).

### Dietary plant-derived mir-146a does not significantly alter gut microbial communities in KO mice

The transition from chow to a plant-based diet resulted in a significant increase in dietary fiber that drove longitudinal shifts in microbial community structure from days 0 to 21. We reasoned that this sharp increase in fiber content could overshadow important microbial changes directed by dietary mir-146a and modified our study design to normalize the baseline diet and minimize large fluctuations in dietary content. To more precisely define the impact of dietary miR-146a on the gut microbiome, we fed KO mice (*n* = 6) vector control diets for 21 days to adjust to the higher fiber content in baseline samples. After 21 days on the vector diet (day 0), mice were split into two groups (*n* = 3/group) and either remained on vector or transitioned to 146a for another 21 days. Fecal samples were collected from each mouse at day zero (after 21 days on vector diet) and at day 21. No distinct differences in health, weight, or behavior Were noted between mouse diet groups during the experiment.

**Fig. 3:**
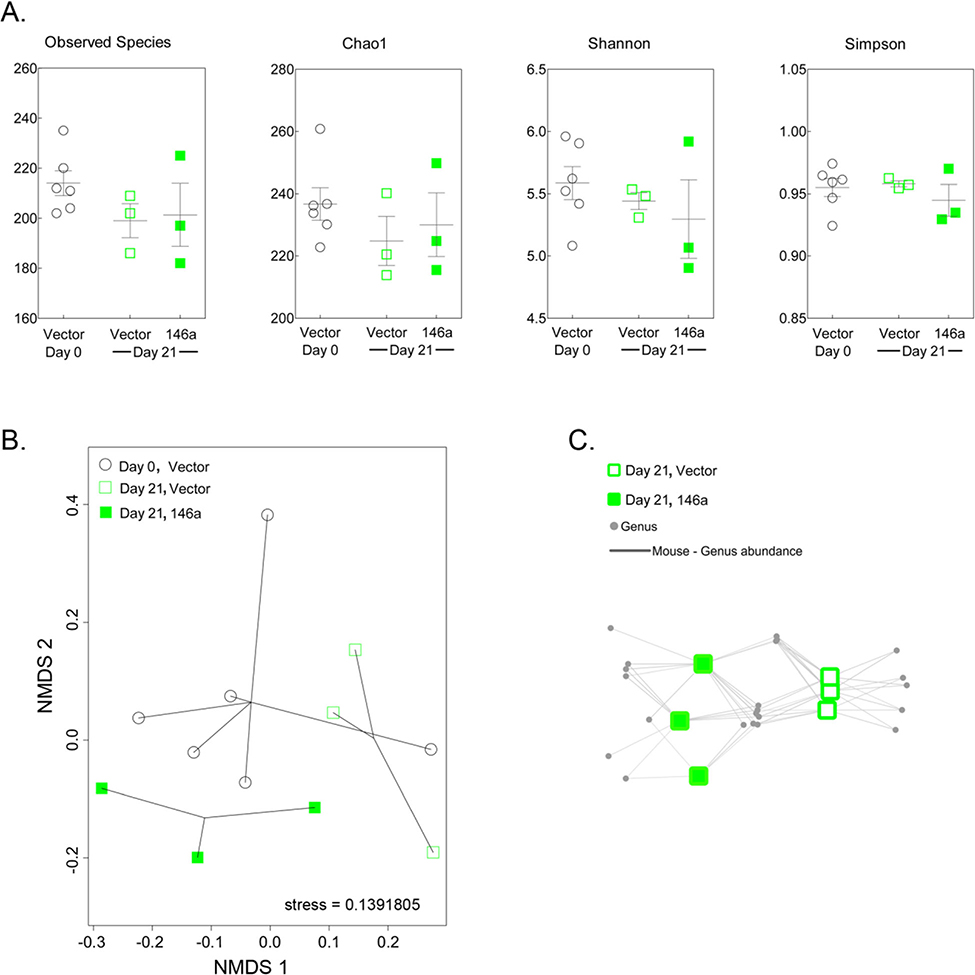
Dietary supplementation with mir-146 in plants does not significantly modulate gut microbiome structure. **a** Alpha-diversity measures of microbiome sequence data from KO mice fed baseline vector diet that transitioned to either vector or 146a diets. **b** Beta-diversity analysis of microbiome sequence data determined using the Bray-Curtis dissimilarity measure plotted with nonmetric multidimensional scaling (NMDS). **c** Bipartite network profile of mice on plant-based vector diet (open green squares) or plant-based 146a diet (full green squares) at 21 days

Minimizing large fluctuations in dietary fiber successfully normalized baseline microbiome communities. Alpha-diversity indicated no difference between mouse gut microbiomes at days 0 and 21, or associated vector or 146a diet at day 21 (Fig. 3a). While dietary clusters could be distinguished in Bray-Curtis dissimilarity plots (Fig. 3b), ANOSIM confirmed that there were no significant differences in the gut microbiomes of KO mice between days 0 and 21 or between vector and 146a diets at day 21 (*R* = 0.1725, *p* = 0.142). Genera abundance networks identified OTUs associated with 146a diets at 21 days, however these associations were not significant (Fig. 3c).

### Transgenic Arabidopsis does not package miR-146a in exosome-like particles

**Fig. 4:**
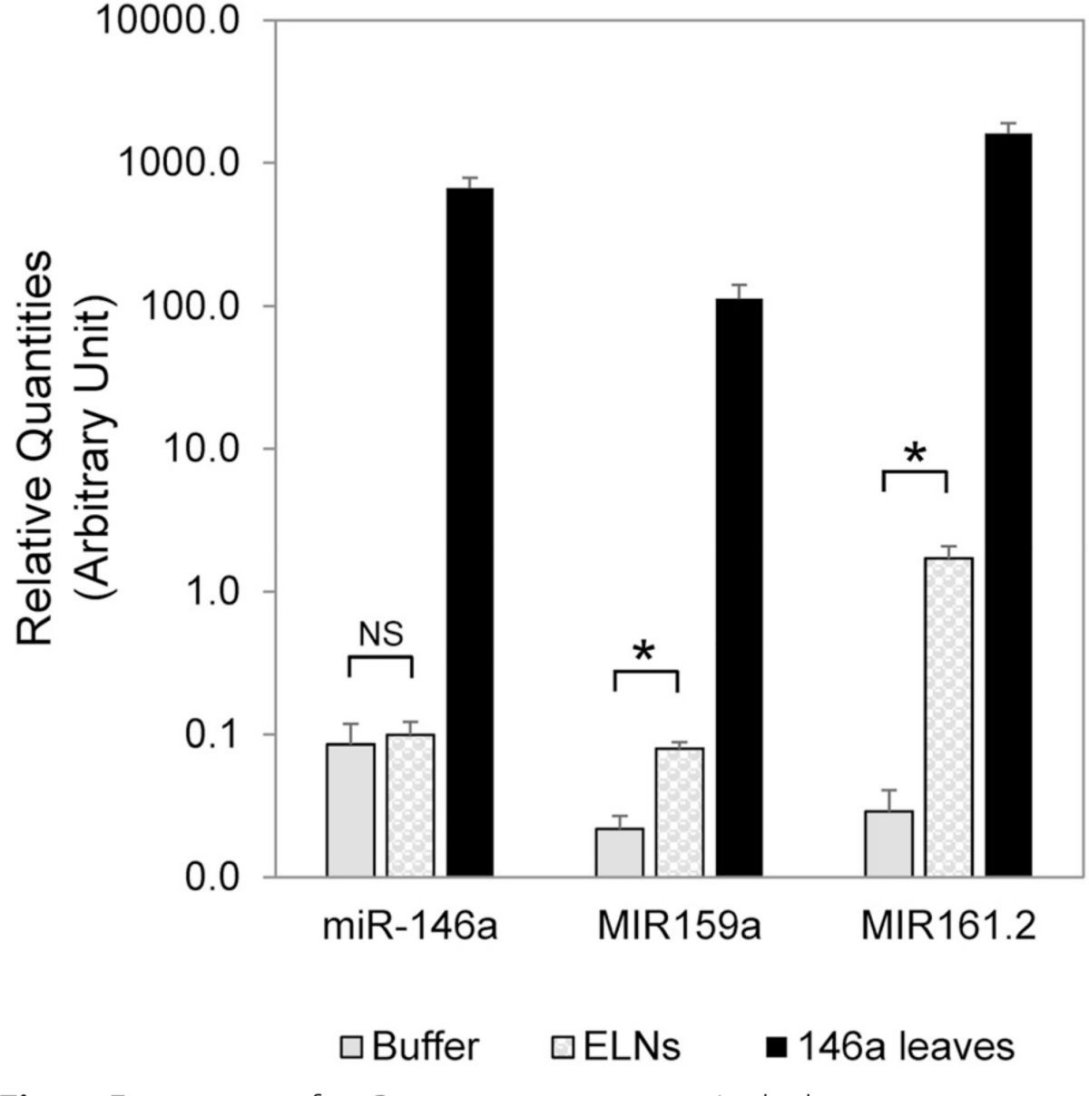
Expression of miR-146a in transgenic Arabidopsis is not associated with ELNs. Quantitative RT-PCR using primers specific for miR-146a, or control RNAs MIR159a and MIR161.2 to amplify corresponding miRNAs from either the miR-146 Arabidopsis leaves (146a leaves), ELNs isolated from the leaves (ELNs), or the ELN resuspension buffer (Buffer). Both MIR159a and MIR161.2 are endogenous plant miRNAs and are found to be associated with ELNs [26]. NS: not statistically significant. Asterisk: *p* < 0.01

The inability of dietary miR-146a to significantly impact the gut microbiome may be due to inefficient packaging into exosome-like nanoparticles (ELNs). Recently, a model has been proposed that each plant has unique ELNs which impact bacteria within the gut [14]. To test the packaging of miR-146a, we first confirmed our previous work and demonstrated that plant-based miR-146a is expressed at relatively high levels in transgenic Arabidopsis (Fig. 4) [23]. We isolated ELNs from our Arabidopsis as done previously [27, 28] and demonstrated by qPCR that endogenous miRNAs MIR159a and MIR161.2 from plants [26] associated with the ELNs, but the murine-derived miR-146a did not (Fig. 4).

## Discussion

MiRNAs can impact gene expression and the concept that dietary plant-derived miRNAs could be bioavailable during GI transit to interact with consumer microbiota is transformative; however, the multitude of different miRNAs found in plant-diets creates a daunting obstacle to ascribe microbiome functionality to a single dietary RNA. In this study, we controlled the diet and consumer interaction in order to precisely investigate the contribution of a single dietary miRNA to microbiome diversity (Supplemental Figure 1).

In our study, plant-based diets were identical with the exception of the expression of mammalian miRNA miR-146a in a genetically modified plant which was confirmed by our bioinformatic analysis (Supplement Table 1) and nutrient analysis of the diet (Table 1). The consuming mice were engineered to be deficient in miR-146a, and established assays document that this deficiency alters microbiome populations. Both the test and control plant diets altered the microbiome consistent with an increase in dietary fiber. The miR-146a diet contributed to modest changes in the microbiome driven by increases in OTUs representing the *Bacteroides* genera and the *Prevotellaceae* family. The changes in these OTUs may represent microbiome fine-tuning by miR-146a that could be beneficial. For example, members of the *Prevotellaceae* family are associated with anti-inflammatory effects and can inhibit growth of other fiber-consuming microbes, while specific *Bacteroides* spp. like B. *thetaiotaomicron* have been linked to vegetarian diets [29]. While these changes were modest, they were statistically significant regardless of the relatively small number of mice used in this study.

Follow-up experiments designed to address concerns that the fiber-driven changes in microbiome composition would hinder our ability to discern subtle differences attributable to a single miRNA did not reveal significant differences in the gut microbiota of mice fed vector or 146a diets. In fact, the differences noted were not statistically significant and did not mimic what had been observed when comparing a baseline chow diet to plant-based diets. The follow-up experiment used few animals and it is possible that significant differences would be observed in larger experiments with greater numbers of animals. These data do support, however, the relative identity of the two plant-based diets in nutritional composition and suggest that dietary delivery of miR-146 is insufficient to overcome the intestinal architectural deficiencies characteristic to miR-146a knock-out mice that must be driving the microbiome community structure in these KO mice.

The fact that miR-146a was not in the plant exosomes (Fig. 4) could impact the delivery of miR-146a to gut microbes and the sera of consumers (Supplemental Figure 3). Furthermore, the results may be different if the miRNA was expressed at higher levels in the plant. This lack of exosome packaging has also been noted in other transgenic miRNAs expressed in plants [23] and highlights a current shortcoming in the field: the inability to selectively bundle transgenic miRNAs inside plant exosome like particles. This limitation is also confounding the ability to ascertain the bioavailability of milk-based miRNAs. Milk-derived miRNAs are speculated to be involved in the “epigenetic priming” of the baby [30]. However, when newborn miR-375 and miR-200c/141 knockout mice received milk from wild-type foster mothers there has been no detectable dietary transfer of these miRNAs [31, 32]. One explanation for this lack of bioavailability is that the miRNAs are not found in the exosomes of the foster mother’s milk.

Additional assays such as alterations in gene expression and sensitivity to pathogens could be used to more extensively monitor intestinal health in these mice. Furthermore, flow cytometry could examine immune cell populations in the intestine that differ between control and miR-146a deficient mice. Previous work has identified taxonomic patterns that correlate with cage effects, age and frailty in the mouse microbiome. Additionally, the mouse microbiome may be more elastic prior to weaning [33] and dietary studies could induce more dramatic changes in younger mice. Future experiments will be done feeding the diets to breeding mice and looking at the microbiome in the progeny. Dietary induced changes of the gut microbiome do not require a great deal of time. Here we have conducted a 21-day feeding trail, but some studies have noted microbiome changes after 7 or 14 days [34]. Accumulating evidence suggests that plant-based miRNAs are not directly involved in consumer gene regulation [3, 35]. Other models suggest complex and highly specific interactions among plant ELNs and distinctive gut bacteria [9].

## Conclusions

Our work here puts forth a model of dietary delivery of plant miRNAs that is straightforward and testable. Dietary plant-based miRNA need not be packaged inside ELNs nor transported systemically to tissues. The effects of these miRNAs are within the gut, where they mirror interactions between plant ELNs and soil microbes [26, 36]. These dietary miRNAs do not have global effects on the GI microbiome structure and composition; they may simply fine-tune microbial communities. Future work will address the mechanisms of plant based dietary miRNA uptake by bacteria and its impact on bacterial gene regulation as a means to modify the microbiome through plant-based diets.

## Methods

### Generation of transgenic plant lines

Transgenic plant lines differentially expressing a single murine miRNA, mmu-miR-146a, were generated for diet studies. Briefly, the binary construct used for overexpression of mmu-miR146a (5′-UGA GAA CUG AAU UCC AUG GGU U – 3′) in the transgenic Arabidopsis line was constructed previously by us [24, 37] and transgenic Arabidopsis lines were grown as described [38]. Homozygous transgenic lines were distinguished and selected using qRT-PCR from plant shoot material as previously described [23, 39].

### Plant diet preparation

Transgenic plant diets were prepared for mouse feeding studies as previously described [10, 15]. Briefly, harvested plants were freeze dried to 30% of fresh weight and finely ground and mixed with chow. The plant-chow diets were prepared by mixing finely ground chow, plant material, and water at 2:1:2 weight ratios, kneaded into 5 g pellets, and stored at − 20 °C until use. Each day 5 g of the plant-chow diet that contained 1 g of dried plant material was fed to each mouse. The daily intake of plant material per each mouse is equivalent to approximately 3.3 g of fresh plant tissue weight. Vector control (vector) and mmu-miR146a (146a) diets differed only by the plant material included in the diet. The wild-type Arabidopsis line expressing empty vector was used to generate vector diets while the 146a diets used transgenic Arabidopsis plants expressing mmu-miR146a.

### Predicted targets of mouse miR146a in Arabidopsis

Targets of mouse mmu-miR146a (5′-UGAGAACUGAAUUCCAUGGGUU-3′) in Arabidopsis (transcript, miRNA genes removed, TAIR10, 2010_12_14 release) were predicted using psRNATarget server (2017 release). The main parameters for the prediction algorithm were set as follows: maximum expectation 5.0, penalty for extending gap 0.5, weight for seeded region 1.5, seed region 2–13, number of mismatch allowed in seed region 1.

### Dietary component analysis

Fifty grams of vector and miR-146a diets were sent to Nestlé Purina Analytical Laboratories (824 Gratiot, St. Louis, MO 6312) for dietary component analysis. Moisture, protein, crude fat, dietary fiber (insoluble and soluble), and ash assays were used.

### Extracellular vesicle isolation

Extracellular vesicles were isolated from transgenic plants and examined for miRNA content. In brief, vesicles were isolated from the apoplastic wash of the vector control and transgenic miR-146a Arabidopsis lines as previously described by Dr. Roger W. Innes [17]. Whole rosettes were harvested at the root and vacuum infiltrated with vesicle isolation buffer (20 mm MES, 2 mmCaCl2, and 0.1 m NaCl, pH 6). Infiltrated plants were blotted to remove excess fluid, placed inside 30-mL syringes, and centrifuged in 50-mL conical tubes at 700 g for 20 min at 4 °C (JA-14 rotor, Avanti J-20 XP centrifuge; Beckman Coulter). The apoplastic wash was filtered through a 0.22-μm membrane and centrifuged successively at 10,000 g for 30 min, and 40,000gfor 60 min at 4 °C. The vesicle pellet was resuspended in Tris-HCl buffer (10 mM, pH 7.5).

### RNA extraction and qRT-PCR analysis of miRNA levels

The extraction of total RNAs from plant tissues was performed using Trizol (ThermoFisher, Waltham, MA). Total RNAs from ELN samples were extracted using miRNeasy Mini Kit from Qiagen (Germantown, MD) following manufacturer’s recommendations. Taqman microRNA Assays for mmu-miR146a, ath-MIR159a, and ath-MIR161.2 were obtained from ThermoFisher (Waltham, MA). One μg of total RNAs per each plant tissue sample or total RNAs from ELN samples equivalent to 1 g of fresh plant leaves were used in each reverse transcription (RT) reaction. Of the 10 μL RT product, 0.5 μL was used for each triplicated quantitative polymerase chain reaction (PCR). qRT-PCR was performed using a Biorad CFX96 Real-Time PCR Detection System, and data were analyzed using Biorad CFX software. Delta-Delta-Ct method was used to calculate relative levels of miRNAs.

### Animal studies

Mouse feeding studies were conducted under protocol AN-2624 approved by the Institutional Animal Care and Use Committee of Baylor College of Medicine (BCM). The miR-146a^−/−^ deficient mice [40] were kindly gifted by Dr. Joel R. Nielson at BCM and were bred and propagated in-house. To genotype the miR146a knockout mice, PCR was done using primers that recognize a region spanning the loxP site: miR146a-F 5′- CTG AGT GGT TCT TGC TGC TG – 3′, miR146a-R 5′- GGA AAT CAC TGC TTG GCA AT – 3′. The PCR conditions were: 94 °C for 3 min; 94 °C for 30 s, 55 °C for 30 s, 72 °C for 30 s, for 34 cycles; 72 °C for 10 min. The wild-type allele is about 150 bp and KO allele is about 250 bp. Heterozygote samples yield both bands. Male and female KO mice (7–8 w.o.) were used in the 21 day feeding studies as previously described [32, 41]. Stool was collected at days 0 and 21 and stored at − 80 °C.

Blood was collected via cardiac puncture as previously described [23]. Sera were separated at room temperature followed by centrifugation to remove all blood cells and debris. Total RNA was extracted from 100 μL of sera using the miRNEASY Kit following the manufacturer’s recommendations [23]. The analysis of miR146a from the blood was done as previously described [23]. Mice were euthanized at study completion and histology was conducted as previously described [42].

### 16S rRNA gene sequencing and data processing

Microbial DNA extraction and 16S rRNA gene sequencing was carried out as described by the Human Microbiome project [43]. Briefly, fecal pellets were processed through the standard MO BIO PowerSoil extraction kit protocol (MO BIO Laboratories, Carlsbad, CA). Amplification and sequencing of the V4 regions of the 16S rRNA gene were performed using the NEXTflex™ 16S V4 Amplicon-Seq Kit 2.0 (Bioo Scientific, Austin, TX) with 20 ng of input DNA, and sequences generated on the Illumina MiSeq platform (Illumina, San Diego, CA) with a minimum of 15,000 sequences generated per sample. Sequence data was processed through the LotuS pipeline [44]. De-multiplexed, quality filtered reads were clustered into operational taxonomic units (OTU) utilizing a modified version of the UPARSE algorithm [45]. Taxonomic assignment was carried out with the RDP classifier and SILVA [46] as the selected database. Organisms potentially classified to the genus level will be based on individual OTUs of significance. OTUs failing to classify as bacteria at the kingdom level were removed prior to further analysis of the dataset. Bacterial diversity, evenness, richness, and relative abundance of the OTUs identified in each sample were evaluated using QIIME [47]. ANOSIM within MOTHUR [48] was be utilized to compare between groups to determine significance.

### Network analysis

Genera relative abundance profiles derived from OTUs clustered as outlined above were visualized as a bipartite network using Cytoscape [49] (version 3.7.1). The genera and mice were represented as nodes and the relative genera abundances as edges. The bipartite networks were generated with the Edge-weighted Spring Embedded Layout algorithm using the abundances as weights. Lower abundances were gradually removed to visualize the profiles with the remaining higher abundant genera. The final network included mouse-genera edges representing 5% or higher relative abundances. The mean and the standard deviation with 95% confidence interval were calculated for each genus and diet type. Two sided paired t-tests were calculated for the groups of mice on chow versus vector, chow versus 146a, and vector versus 146a diets.

## Supplementary Material

exRNA supplement **Additional file 1: Figure 1.** Schematic of miR-146a feeding study. Vector and miR-146a diets were fed to 10 KO mice for 21 days. Fecal samples were collected every 7 days. Mice were sacrificed at the end of the feeding study and sections of small intestine prepared for histological analysis. Microbial DNA was isolated from mouse fecal specimens and the V4 region of the 16S rRNA genes sequenced for microbiome analysis.
**Additional file 2: Figure 2.** Characterization of the intestinal epithelium and microbiomes of KO mice fed vector or 146a diets. Small intestinal sections of KO mice on either vector or 146a diets were formalin fixed and stained with haemotoxylin and eosin. Sections (5 μm) were imaged using an Olympus IX70 microscope and a SPOT RT Slider CCD Camera. The images shown are representative of the groups as a whole. KO = knockout mice; Vector = control vector diet; 146a = transgenic miR-146a-expressiong diet; black bar = 50 mm scale.
**Additional file 3: Figure 3.** Serum miR-146a measurement in miR-146a knockout mice fed transgenic miR-146a-expressing Arabidopsis. miR-146a knockout mice (146KO) were fed either plant-based diet containing transgenic Arabidopsis overexpressing the murine miR-146a (miR146OE-diet), or wildtype Arabidopsis containing empty vector (WT-diet). The C57BL/6 control mice were fed chow diet. The sera were collected from the mice after 7 days of feeding. N = 5. ns: statistical difference not significant.
**Additional file 4: Table 1.** Potential targets of murine miR-146a in Arabidopsis thaliana predicted by psRNATarget.

## Figures and Tables

**Table 1:** Percent compositions of vector and miR-146a dietary components

Assay	Percent composition		Units
	Vector	146a	

**Moisture**	44.4	46.0	%
**Protein**	13.5	12.8	%
**Crude fat**	1.48	1.63	%
**Insoluble dietary fiber**	6.58	6.41	%
**Soluble dietary fiber**	< 0.50	< 0.50	%
**Total dietary fiber**	6.70	6.84	%
**Ash**	3.60	3.11	%

## Abbreviations

ANOSIM: Analysis of similarities
BCM: Baylor College of Medicine
ELNs: Exosome-like nano- particles
GI: Gastrointestinal tract
KO: Knockout
miRNA: microRNA
NMDS: Non-metric multidimensional scaling
OTU: Operational taxonomic unit
qRT-PCR: Real-time polymerase chain reaction
WT: Wild-type
