# Supplementary material for: Dietary impact of a plant-derived microRNA on the gut microbiome
Source: ExRNA. Author manuscript; Available in PMC 2021 Feb 3. (PMC7856875; doi:10.1186/s41544-020-00053-2)
Supplement: exRNA supplement — Additional file 1: Figure 1. Schematic of miR-146a feeding study. Vector and miR-146a diets were fed to 10 KO mice for 21 days. Fecal samples were collected every 7 days. Mice were sacrificed at the end of the feeding study and sections of small intestine prepared for histological analysis. Microbial DNA was isolated from mouse fecal specimens and the V4 region of the 16S rRNA genes sequenced for microbiome analysis. Additional file 2: Figure 2. Characterization of the intestinal epithelium and microbiomes of KO mice fed vector or 146a diets. Small intestinal sections of KO mice on either vector or 146a diets were formalin fixed and stained with haemotoxylin and eosin. Sections (5 μm) were imaged using an Olympus IX70 microscope and a SPOT RT Slider CCD Camera. The images shown are representative of the groups as a whole. KO = knockout mice; Vector = control vector diet; 146a = transgenic miR-146a-expressiong diet; black bar = 50 mm scale. Additional file 3: Figure 3. Serum miR-146a measurement in miR-146a knockout mice fed transgenic miR-146a-expressing Arabidopsis. miR-146a knockout mice (146KO) were fed either plant-based diet containing transgenic Arabidopsis overexpressing the murine miR-146a (miR146OE-diet), or wildtype Arabidopsis containing empty vector (WT-diet). The C57BL/6 control mice were fed chow diet. The sera were collected from the mice after 7 days of feeding. N = 5. ns: statistical difference not significant. Additional file 4: Table 1. Potential targets of murine miR-146a in Arabidopsis thaliana predicted by psRNATarget. [file NIHMS1645880-supplement-exRNA_supplement.pdf]

Supplemental Figure 1. Schematic of miR-146a feeding study. Vector and miR-146a diets were fed to 10 KO mice for 21 days. Fecal samples were collected every 7 days. Mice were sacrificed at the end of the feeding study and sections of small intestine prepared for histological analysis. Microbial DNA was isolated from mouse fecal specimens and the V4 region of the 16S rRNA genes sequenced for microbiome analysis.

Supplemental Figure 2. Characterization of the intestinal epithelium and microbiomes of KO mice fed vector or 146a diets. Small intestinal sections of KO mice on either vector or 146a diets were formalin fixed and stained with haemotoxylin and eosin. Sections (5 µm) were imaged using an Olympus IX70 microscope and a SPOT RT Slider CCD Camera. The images shown are representative of the groups as a whole. KO = knockout mice; Vector = control vector diet; 146a = transgenic miR-146a-expressiong diet; black bar = 50 mm scale.

Supplemental Figure 3. Serum miR-146a measurement in miR-146a knockout mice fed transgenic miR-146a-expressing Arabidopsis. miR-146a knockout mice (146KO) were fed either plant-based diet containing transgenic Arabidopsis overexpressing the murine miR-146a (miR146OE-diet), or wildtype Arabidopsis containing empty vector (WT-diet). The C57BL/6 control mice were fed chow diet. The sera were collected from the mice after 7 days of feeding. N = 5. ns: statistical difference not significant.

Supplemental Table 1. Potential targets of murine miR-146a in *Arabidopsis thaliana* predicted by psRNATarget.

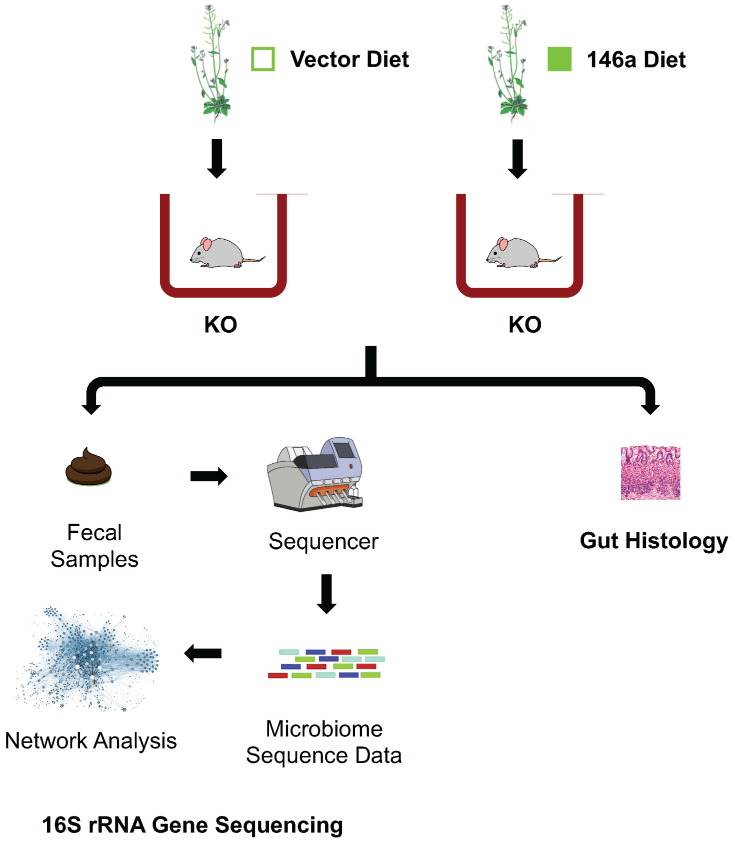

Supplemental Figure 1

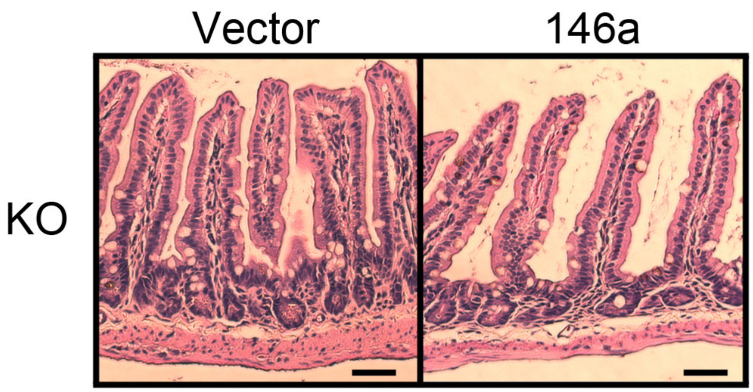

Supplemental Figure 2

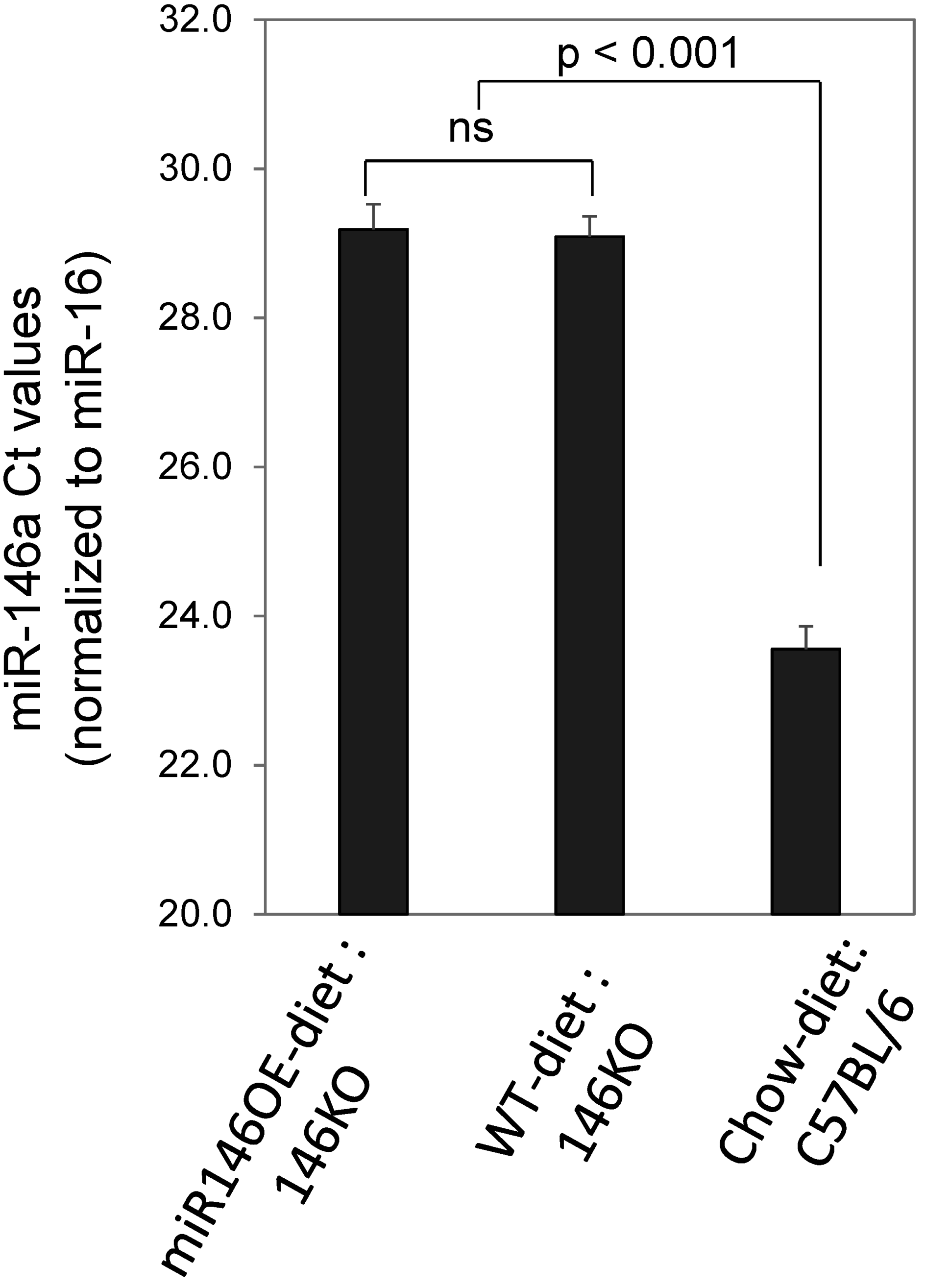

Supplemental Figure 3.

Supplemental Table 1. Potential targets of murine miR-146a in *Arabidopsis thaliana* predicted by psRNATarget.

Target Gene ID	Alignment	Target Description
AT3G24880	miRNA 22 UUGGGUACCUUAAGUCAAGAGU 1<br>...:: :::::::::::.:::<br>Target 3543 UGUUCACGGAAUUCAGUUUUCA 3564	| Symbols: | Helicase/SANT-associated, DNA binding protein | chr3:9086242-9095537 REVERSE LENGTH=6089
AT5G07980	miRNA 22 UUGGGUACCUUAAGUCAAGAGU 1<br>::::: ::::::::::.:..<br>Target 4712 UCCCCAUAGAAUUCAGUUUUUG 4733	| Symbols: | dentin sialophosphoprotein-related | chr5:2549166-2555519 REVERSE LENGTH=4966
AT2G06780	miRNA 22 UUGGGUACCUUAAGUCAAGAGU 1<br>::.::: ::::::::: :::::<br>Target 1360 AAUCCAAGGAAUUCAGAUCUCA 1381	| Symbols: | transposable element gene | chr2:2709951-2712872 REVERSE LENGTH=2922
AT4G22860	miRNA 22 UUGGGUACCUUAAGUCAAGAGU 1<br>:::. ::::.::.::::.:::<br>Target 1568 UACCUUUGGAGUUUAGUUUUCA 1589	| Symbols: | Cell cycle regulated microtubule associated protein | chr4:11997393-12001631 FORWARD LENGTH=2222
AT4G11990	miRNA 22 UUGGGUACCUUAAGUCAAGAGU 1<br>::: :::..::.::::.:::<br>Target 1391 CUCCCUUGGGGUUUAGUUUUCA 1412	| Symbols: | Cell cycle regulated microtubule associated protein | chr4:7186096-7189875 REVERSE LENGTH=1913
